# Total Coumarins from* Hydrangea paniculata* Protect against Cisplatin-Induced Acute Kidney Damage in Mice by Suppressing Renal Inflammation and Apoptosis

**DOI:** 10.1155/2017/5350161

**Published:** 2017-03-06

**Authors:** Zhang Sen, Ma Jie, Yang Jingzhi, Wang Dongjie, Zhang Dongming, Chen Xiaoguang

**Affiliations:** State Key Laboratory of Bioactive Substances and Functions of Natural Medicines, Institute of Materia Medica, Chinese Academy of Medical Sciences and Peking Union Medical College, Beijing 100050, China

## Abstract

*Aim.* Hydrangea paniculata (HP) Sieb. is a medical herb which is widely distributed in southern China, and current study is to evaluate renal protective effect of aqueous extract of HP by cisplatin-induced acute kidney injury (AKI) in animal model and its underlying mechanisms.* Materials and Methods.* HP extract was prepared and the major ingredients were coumarin glycosides. AKI mouse models were established by single i.p. injection of 20 mg/kg cisplatin, and HP was orally administrated for total five times. The renal biochemical functions, pathological staining, kidney oxidative stress, and inflammatory status were measured. Apoptosis of tubular cells and infiltration of macrophages and neutrophils were also tested.* Results.* HP administration could improve the renal function by decreasing concentration of blood urea nitrogen (BUN) and creatinine and attenuates renal oxidative stress and tubular pathological injury and apoptosis; further research demonstrated that HP could inhibit the overproduction of proinflammatory cytokines and regulate caspase and BCL-2 family proteins. HP also reduced renal infiltration of macrophages and neutrophils, and its effect might be by downregulating phosphorylation of ERK1/2 and stat3 signaling pathway.* Conclusions.* This present study suggests that HP could ameliorate cisplatin induced kidney damage by antioxidation and suppressing renal inflammation and tubular cell apoptosis.

## 1. Backgrounds

Cisplatin is used as one of the most effective cytotoxic drugs for the treatment of different cancers like head and neck cancer, non-small-cell lung cancer, ovarian cancer, and testicular cancer [1, 2]. Though the potency of the drug is highly effective, its serious nephrotoxic nature as adverse side effect restricts its long-term use and effective dose selection in cancer chemotherapy [3, 4]. Several possible therapeutic strategies have been reported to ameliorate the cisplatin-induced renal injury in several experimental animal models, but are not effective in the clinical practice, as the use of saline in vigorous hydration is still most-adopted option in clinical practice [1, 5]. Therefore, screening of novel agents which are effective or combination methods is highly essential in preventing cisplatin-induced renal injury.

Currently, the mechanisms which are possibly involved in cisplatin-induced nephrotoxicity could be direct cytotoxicity effect on renal tubular epithelial cells [6], induction of apoptosis [7], dysregulation of cell-cycle proteins [8], oxidative stress [9], inflammation [10], and also many other unknown mechanisms. Several reported studies have shown that increase in release of proinflammatory mediators (chemokines and cytokines) and infiltration of macrophage and neutrophils into damaged kidney tissues are the key pathophysiologic roles in cisplatin-induced acute kidney injury (AKI) [11, 12]. Cisplatin activates TNF*α*, which plays key role in proinflammatory chemokines and cytokines network in the kidney. Blocking TNF*α* function plays a protective role against cisplatin-induced nephrotoxicity [13]. Besides TNF*α*, cisplatin-induced nephrotoxicity is aggravated by overproduction of IL1*β* [14] and IL-6 and overphosphorylation and hyperactivation of extracellular regulated protein kinases 1 and 2 (Erk1/2) and stat3 [15]. Therefore, modulating renal inflammatory reaction in post-cisplatin treatment might reduce cisplatin-induced renal injury.

Cisplatin-induced apoptosis in tubular epithelial cells is another important pathological factor contributing to AKI. Activation of caspase family members and inactivation of BCL family members play an important role in mediating apoptosis. Role of activated caspase family (caspases 3, 7, and 8) is widely known for its involvement in the cisplatin-induced tubular cell apoptosis and reduction in renal injury could be seen by inhibition of caspase family [6, 16].

Based on abundant of documents on traditional Chinese medicine, hundreds of medicinal plants have shown remarkable anti-inflammatory activity.* Hydrangea paniculata *(HP) Sieb. is one of them and widely distributed in southern region of China.* H. paniculata* is documented as local folk medicinal prescription in Yun Nan province of China. Its beneficial effect is that its stem has bioactivity against inflammation, malaria, and fever [17]. However, unlike some well-known medical herbs, such as* Panax ginseng *C. A. Mey. and* Hedyotis diffusa *Wild, the English literature research publication about HP usage is limited, and the related documents are often only in Chinese literatures in Chinese. In recent years, the chemists from our institute conducted several preliminary studies to identify and explain the major constituents isolated from* H. Paniculata*, and most of these constituents were related to anti-inflammation and antioxidation effect, which shows its possible role in renal protection effect because many acute and chronic kidney injuries are caused by system inflammation, oxidation, and autoimmunity.

The 20% ethanol-eluted fraction from the stem of* H. paniculata *had high concentration of coumarin glycosides. Skimmin and apiosylskimmin were the major constituents along with other coumarin glycosides. Our previous studies showed that single skimmin administration can slow down the progression of streptozocin-induced diabetic nephropathy [18] and BSA-induced membranous glomerulitis [19]. DL-galactosamine-induced toxicity in HL-7702 cells can be downregulated by hepatoprotective activity of some coumarin glycosides [20]. Serum deprivation-induced PC12 cell damage was shown to be ameliorated by neuroprotective effects of various coumarin glycosides [21]. Besides coumarins, HP also contains two major iridoids, loganin and sweroside; both were known for its anti-inflammation activity [22, 23].

The present study investigates the protective effect of* H. paniculata* extract in kidney, against renal function deterioration, inflammation, oxidation status, and apoptosis caused by cisplatin and its underlying mechanisms.

## 2. Materials and Methods

### 2.1. Preparation of Extract of* Hydrangea paniculata*

*Hydrangea paniculata* Sieb. (Saxifragaceae) stems were procured in May 2014 from Jinxiu County, Guangxi Zhuang Autonomous Region, China. Mr. Guangri Long from the Liuzhou Forestry Bureau of Guangxi identified the collected samples. A sample specimen with ID 4645 was deposited in Institute of Materia Medica, Chinese Academy of Medical Sciences, Beijing, China.

### 2.2. Preparation of Plant Samples

The stems of* H. Paniculata* (10 Kg) were air dried and powdered and then extracted with H_2_O for three times each for 3 h. The whole extract was with H_2_O and 20% EtOH by passing through macroporous resin column (HPD100). The ethanol portion was dried using a rotary vacuum evaporator and the residue was refluxed with 95% EtOH. After EtOH evaporation in vacuum, the remaining aqueous part (700 g) was eluted with water and 20% EtOH by passing it through a macroporous resin column (HP2MGL). The 20% ethanol fraction was evaporated under reduced pressure to obtain the sample. Finally, the obtained dry sample per gram was equivalent to 91 g raw medical herbs.

### 2.3. Determination of the Content of Main Constitutes

*HPLC Condition.* To analyze glycosides, the mobile phase used contains 88% (v/v) water (A) and 12% (v/v) methanol (B). The flow rate was set at 1.0 mL/min with the column temperature at 35°C. The detection wavelength was 318 nm. The main chemical constituents of the product were coumarins and iridoids. The determination of their content was performed on HPLC condition as described above. The determination and quantification of two major coumarins, skimmin and apiosylskimmin, were shown in Figure 2. They were determined by HPLC with standards purchased from Guilin Huiang Biochemistry Pharmaceutical Company Ltd (China).

### 2.4. Animals

Thirty adult male C57BL/6 mice (18–20 g) aged 6–8 weeks were procured from the Beijing SPF Laboratory Animal Technology Co., Ltd (Beijing, China). The animals were supplemented with adequate food and water ad libitum in pathogen-free facility, maintained at constant temperature (22 ± 2°C) and illuminated from 7:00 a.m. to 7:00 p.m. All animal experiments were approved by the Ethics Committee of Laboratory Animals of Beijing Municipality and were performed in accordance with relevant guidelines and regulations (ethic approval number IMM-2016-1988).

### 2.5. Experimental Design and Measurement of Renal Function

**Figure 1:**
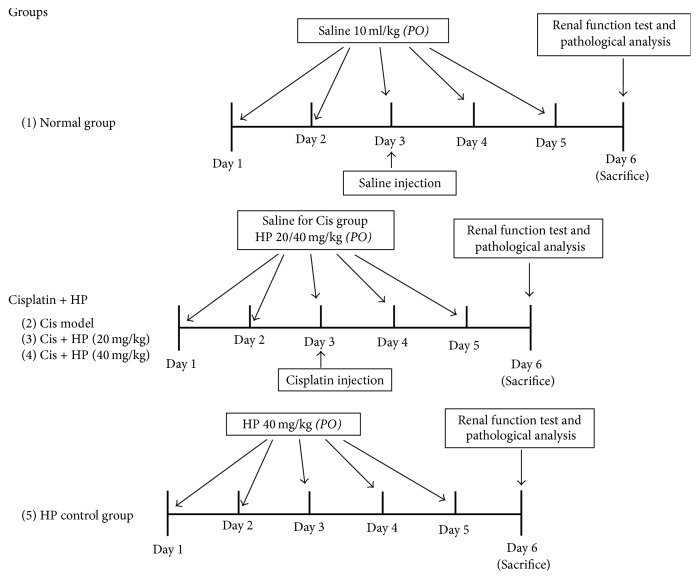
Experimental design and animal group classification.

The water and 20% ethanol* H. paniculata *extract was termed as “HP” in the present study. Thirty mice were divided randomly with six mice in each of the five groups as follows: the first group was normal control group (NC; *n* = 6) that received intraperitoneal (i.p.) injection of vehicle solution (0.9% saline; 10 ml/kg); the second group was cisplatin model group (*n* = 6) that received a single* i.p.* injection of cisplatin (20 mg/kg) dissolved in 0.9% saline and treated orally with vehicle buffer; the third and fourth group were orally given HP at dose of 20 and 40 mg/kg, respectively, with cisplatin injection; and the fifth group was only given HP 40 mg/kg orally for five consecutive days as control to assess the HP itself toxicity. The experimental procedure was as follows: vehicle buffer or HP was pretreated orally for two days earlier for model group and HP treatment groups, respectively, and on third day, the animals from these three groups received single* i.p.* injection of 20 mg/kg cisplatin. After that, it was continued by oral administration of HP or vehicle buffer for another consecutive three days. HP was dissolved in the 0.9% saline and was effectively soluble in it. The illustration of animal experimental procedure was shown in Figure 1.

Three days after cisplatin dosage, blood was collected from the eye endocanthion in all mice before all the mice were sacrificed by cervical dislocation. The sera samples were collected from blood by centrifugation at 1,500*g* in 4°C for 15 min and stored. BUN and serum creatinine (Scr) levels were measured using commercial kits (Beijing Beihua Biotechnology, Beijing, China). Kidneys from each animal were weighed to calculate the relative kidney weight. Relative kidney weight was calculated as the percentage weight of kidneys divided by the final body weight of the mice. The kidneys were excised immediately and cut into half by coronal position after weighing. For protein extraction, a half of each excised kidney was stored at −80°C, and the remaining sections were fixed in buffered paraformaldehyde (4%) at 4°C and then embedded in paraffin and some were immediately frozen by liquid nitrogen for preparing frozen tissue sections.

### 2.6. Histopathologic Observation

In kidney, the histopathological changes were observed by Hematoxylin and Eosin (HE) staining. Kidney samples embedded in paraffin were sectioned into 2 *μ*m thick sections. HE reagent was used for staining the deparaffinized sections. Tubular cell damage was analyzed by average index of renal tubular necrosis from one section in 10 different fields. The scoring standard was recorded as follows: 0 indicated no damage, 1 indicated less than 25% damage, 2 indicated 25–50% damage, 3 indicated 50–75% damage, and 4 indicated more than 75% damage. Tubular necrosis was defined as the loss of the proximal tubular brush border, protein cast formation, blebbing of apical membranes, and tubular epithelial cell detachment from the basement membrane. Two independent experienced pathologists performed morphometric examination in a blinded manner.

### 2.7. Analysis of Tubular Cell Apoptosis by TUNEL Assay and DAPI Staining

Terminal deoxynucleotidyl transferase dUTP nick end labeling (TUNEL) assay was used for detecting DNA fragmentation in frozen kidney tissue sections (3 *μ*m) by using In Situ Cell Death Detection Kit, Fluorescein (Roche, USA). Briefly, the frozen tissue slides were fixed by 4% fresh-prepared paraformaldehyde solution for half an hour at room temperature at beginning and then washed by PBS buffer for another thirty minutes. The slides were further incubated with ice cold permeabilisation buffer (freshly prepared 0.1% Triton X-100, 0.1% sodium citrate) for two minutes and washed completely by PBS. For the last step, the tissue in the slides was covered by 50 *μ*L TUNEL reaction buffer with fluorescein at 4°C overnight. On the following day, the slides were washed thrice in PBS and mounted by DAPI containing gum. The pictures were observed and evaluated under fluorescence microscopy (Observer A1 inverted microscope, ZEISS, Germany) with 200x actual magnification. Excitation wavelength in the range of 450 nm to 500 nm and detection wavelength range of 515–565 nm were used for TUNEL fluorescence and the DAPI staining was observed with an excitation wavelength of 340 nm. The cells that were positively labeled were quantified in ten different fields per slide by Image pro plus 6.0 software (Media Cybernetics, Georgia, USA).

### 2.8. Tissue Homogenates and Measurement of Oxidative Stress Markers

Kidneys were homogenized in PBS using a Tissuelyser-38 (Jingxin Industrial Development Co., Ltd, Shanghai, China) and then centrifuged at 3000*g* for 15 minutes. The protein content was measured from the supernatant using the bicinchoninic acid (BCA) assay (Applygen Technologies Inc., Beijing, China). Supernatant aliquots were used to determine the kidney oxidative stress by testing the catalase (CAT), glutathione (GSH), activity of superoxide dismutase (SOD), and lipid peroxidation (LPO). The test procedure was exactly followed as the guidance of each commercial kit (Nanjing Jiancheng Biotechnology, Nanjing, China).

### 2.9. Western Blot Analysis for Cytokines, Apoptosis Mediators, and Biomarkers of Macrophage and Neutrophil

The frozen kidney cortex was homogenized and lysed in a RIPA lysis buffer (5 mM EDTA, 10 mM Tris-HCl, 1 mM EGTA, 150 mM NaCl, and 10% Triton X-100) with protease inhibitor (Roche, USA). After centrifugation at 13,000 rpm for 30 min in 4°C, the protein content in the collected supernatant was measured using BCA assay (Applygen Technologies Inc., Beijing, China). Equal amounts of total protein (approximately 30 ug) from each group were boiled for 10 min at 95°C and loaded onto a 10% SDS-polyacrylamide gel electrophoresis. After the gel run, the samples were transferred to polyvinylidene difluoride membranes (PVDF, Millipore Co., Billerica, MA, USA) at 200 mA for 90 min. After washing the membranes in Tris-buffered saline with Tween 20 (TBS/T), PVDF membranes were incubated in 5% skim milk (Difco, BD, USA) at room temperature for 1 h. Then the membranes were washed in TBS/T and the primary antibodies like caspase 3 (1 : 1000 dilution, Cell Signaling Technology, CST, USA), cleaved caspase 3 (1 : 1000 dilution, CST), caspase 7 (CST), TNF-*α* (1 : 2000 dilution, Abcam), IL-1*β* (1 : 1000 dilution, Abcam), BCL-2 (1 : 1000 dilution, CST), BCL-xL (1 : 1000 dilution, CST), FasL (1 : 1000 dilution, Santa Cruz), F4/80 (a mouse macrophage biomarker, 1 : 1000, Abcam), stat3 (1 : 1000 dilution, CST), phosphorylated-stat3 (Tyr705, 1 : 1000 dilution, CST), p44/42 MAPK (Erk1/2) (1 : 1000 dilution, CST), Phospho-p44/42 MAPK (Erk1/2) (Thr202/Tyr204, 1 : 1000 dilution, CST), F4/80 (1 : 1000, Abcam, USA), Ly6G (1 : 1000, Invitrogen, Life Technology), and *β*-actin (1 : 1000 dilution, Santa Cruz) were added and incubated at overnight in 4°C. The membranes were washed thrice with TBST and incubated with HRP-conjugated secondary antibodies (Santa Cruz, USA, 1 : 2000 dilution) at room temperature for 1 h. The membranes were visualized by an enhanced chemiluminescence system (LAS 4000, General Electric, USA). Densitometric analysis was performed by using image J software (CA, USA).

### 2.10. Immunofluorescence of F4/80 and Ly6G in Renal Tissues

Immunofluorescence staining was performed for macrophage biomarker F4/80 and neutrophil membranous biomarker Ly6G. Removed kidney tissues were frozen immediately with liquid nitrogen and then were cut into 2 *μ*m thick frozen sections with the help of freezing microtome (Leica Biosystems, Germany) by skilled technician. Endogenous peroxidase was blocked for 20 min in 3% hydrogen peroxide, and PBS was used to rinse the samples. After blocking with 5% BSA, the slides were incubated at 4°C with a rabbit anti-mouse Ly6G (dilution 1 : 100; Abcam, USA) and F4/80 antibody (1 : 50, Invitrogen, Life Technologies, USA) overnight. The anti-rabbit fluorescein-conjugate second antibody (Invitrogen, Thermo Fisher Scientific, USA) was used to detect and amplify the primary signals by light emission. The pictures were observed and evaluated under fluorescence microscopy (Observer A1 inverted microscope, ZEISS, Germany) with 400x actual magnification. Two observers evaluated the slides, who were unaware of the slides classification. F4/80-positive and Ly6G-positive cell number in each section were calculated by counting positively stained cells in 10 fields per slide at 400x magnification.

### 2.11. Data Analysis and Statistical Methods

For each experimental group (*n* = 6), the results were given as the group mean ± standard error of the mean (SEM). The data was analyzed employing One Way Analysis of Variance (ANOVA) followed by and post hoc Bonferroni test (SPSS Statistical package 19.0, USA). A *P* value < 0.05 is considered statistically significant.

## 3. Results

### 3.1. Determination of Main Ingredients of HP Extract

**Figure 2:**
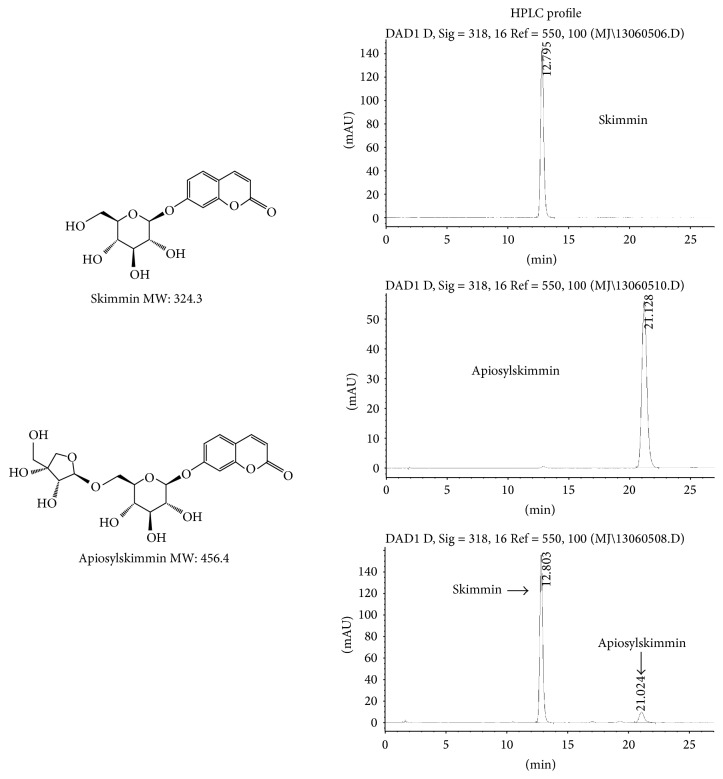
Chemical structure of skimmin and apiosylskimmin and HPLC profile of major coumarins from HP. Standard skimmin and apiosylskimmin were purchased from Guilin Huiang Biochemistry Pharmaceutical Company Ltd (China).

Most ingredients isolated by our specific extraction procedure from* H. paniculata* belong to coumarin family. The most abundant ones are skimmin (35.3%) and apiosylskimmin (18.9%). Besides them, there were other eight structure-known coumarins which account for 25% of all HP ingredients. Total coumarins reach to approximate 80% percent of HP extract. The remaining major constituents were two iridoids, loganin and sweroside, which account for about 15% of HP extract. The chemical structure of skimmin and apiosylskimmin and the HPLC profile of major coumarin derivatives from HP were shown in Figure 2.

### 3.2. HP Can Reduce Renal Dysfunction in Cisplatin-Induced Renal Injured Mice

The serum levels of BUN and creatinine in mice were measured 72 h after cisplatin injection to study the effects of HP. Cisplatin challenge significantly elevated serum BUN (58.6 ± 6.16 versus 29.5 ± 0.72 mg/dl, cis model versus normal control) and creatinine (0.73 ± 0.08 versus 0.47 ± 0.06 mg/dl, cis model versus normal control) levels compared to normal control group (Table 1). BUN and creatinine levels significantly declined after HP treatment compared to cisplatin model control in a dose-dependent manner (Table 1). Cisplatin-treated group also showed that relative kidney weight (1.37 versus 0.65) was significantly increased compared to normal control but downregulated by HP treatment (Table 1). Use of HP alone at 40 mg/kg did not change BUN and Scr levels when compared to vehicle-treated normal group, as well as relative kidney weight.

### 3.3. HP Reduced the Oxidation in the Cisplatin-Induced Kidney Tissues

Cisplatin challenge in kidney tissues caused significant increase in oxidative stress, leading to lower GSH level and higher LPO production, as well as decrease in antioxidant enzyme activities like catalase and SOD (Table 2). HP administration significantly ameliorated the oxidative stress in the cisplatin-challenged kidney tissues by increasing the GSH level, SOD, and catalase activity, with decrease in LPO contents. Application of HP alone did not show pronounced influence on these oxidative biomarkers.

### 3.4. HP Ameliorated the Renal Tubular Impairment on Pathological Analysis

**Figure 3:**
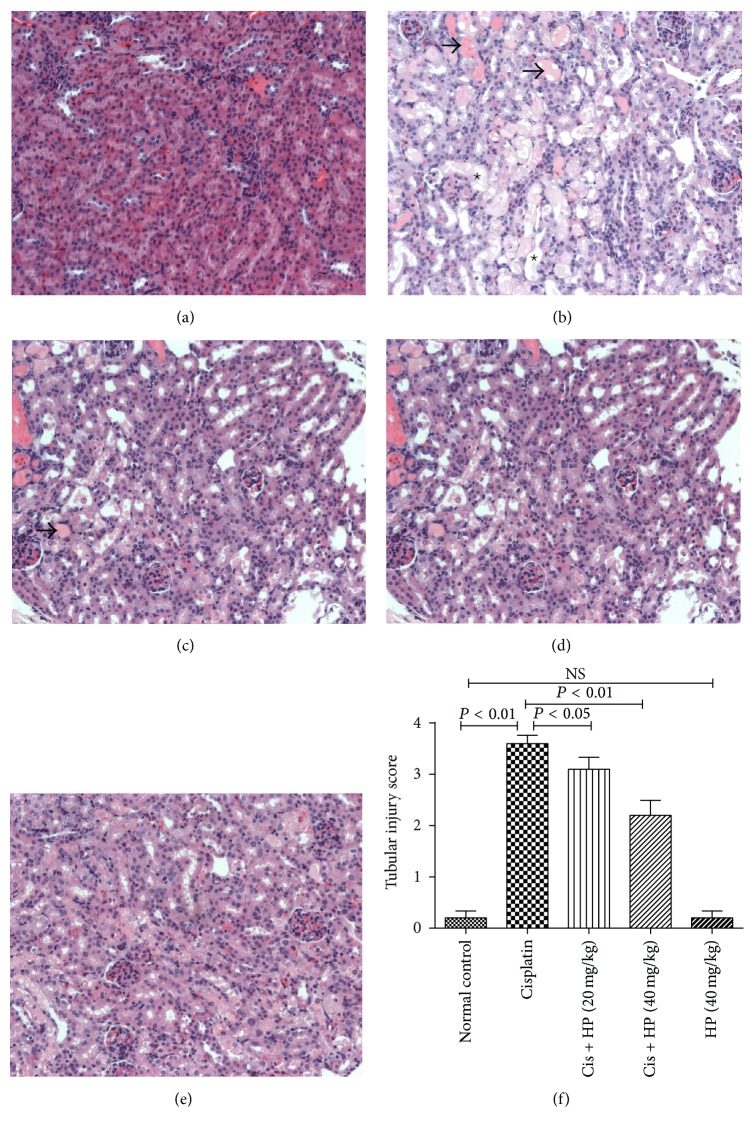
Effect of HP on cisplatin affected renal tissues of mice by light microscopic examination (H&E). (a) Normal group and (b) cisplatin-treated group have severe tubular damage with acute tubular necrosis (dashed thick arrow), wide tubular epithelial vacuolation, apoptotic tubular epithelium, and cast formation (star, ∗). (c) HP with 20 mg/kg and (d) HP with 40 mg/kg and cisplatin-treated group showed significantly less impaired renal tubules with minimal focal vacuolation of the tubular epithelium. (e) HP treated alone group showed normal nonaffected tubular epithelium. (f) Renal injury score. Each renal injury score value represents the mean of six mice. All the pictures were taken under 200x magnification.

HE staining for each kidney sections was performed to analyze the HP effect on renal tubular damage induced by cisplatin under light microscopy (×200 magnification). In cisplatin-treated group, serious tubular injuries were noted. The main pathological characteristics included degeneration of epithelial cells and atrophy, hyaline materials and cast formation in the tubular lumen, and apoptosis and necrosis of tubular cells. After HP treatment, the damage in the tubular epithelial cells was limited to mild necrosis, reduced epithelial swelling, and lesser cast formation (Figures 3(a)–3(d)). HP treatment could significantly reduce the tubular necrosis score dose-dependently when compared to cisplatin-treated mice alone (Figure 3(f)). HP alone did not produce any visible impairment by pathological analysis (Figure 3(e)).

### 3.5. HP Reduced the Apoptosis by TUNEL Assay

**Figure 4:**
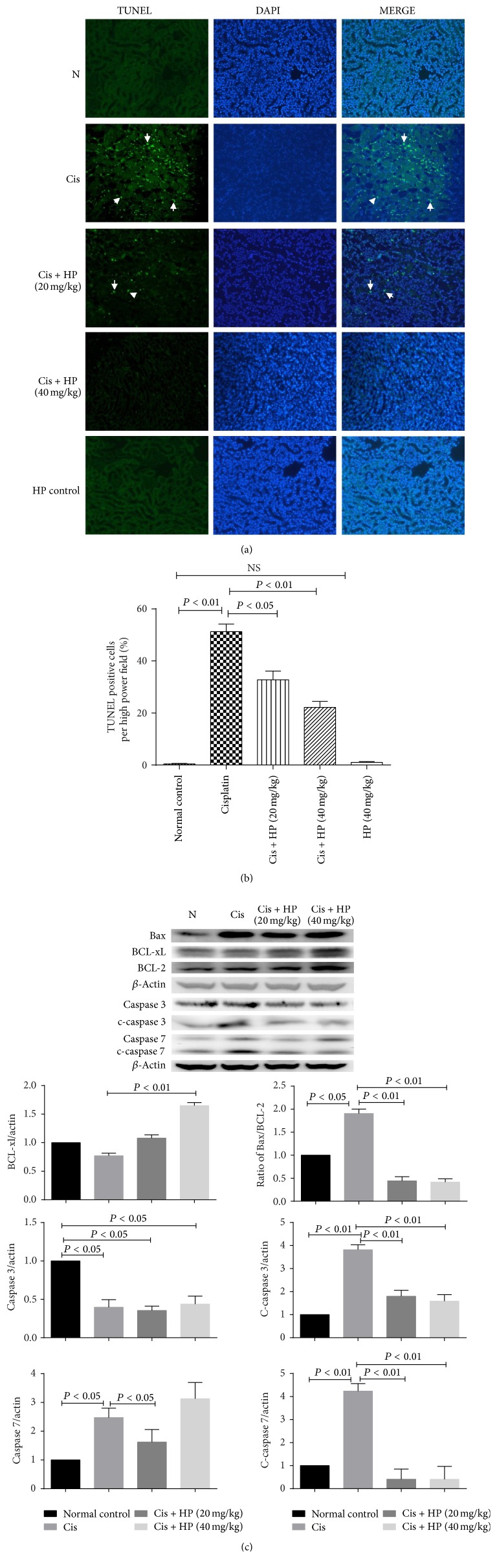
HP protective effect on apoptosis of tubular cells and its effect on Bcl-2 family protein and caspase family protein expression. (a) Effect of HP on cisplatin-induced cell apoptosis of proximal tubules by TUNEL assay with fluorescence microscopy of DAPI staining. Cis model group showed condensed state of nuclei and high abundant TUNEL positive cells (arrows). Treatment with HP reduced TUNEL positive cells, nuclear fragmentation, and condensation. (b) Quantitative analysis of TUNEL positive cells per field. All the pictures were taken under 200x magnification. (c) Western blot was used to evaluate the protein expression of Bax, Bcl-2, Bcl-xL, caspase 3, and caspase 7 among four groups and densitometric analysis was conducted for ratio of Bax/Bcl-2. Expression of Bcl-xL, caspase 3/cleaved caspase 3, and caspase 7/cleaved caspase 7 is presented as the relative ratio of each protein to actin. Data are expressed as mean ± SEM of three independent experiments.

Renal tubular apoptosis is one of important mechanisms seen in acute kidney injury induced by cisplatin [24, 25]. In the current study, we performed TUNEL assay to evaluate the severity in tubular epithelial cell apoptosis. As shown in Figures 4(a) and 4(b), a high amount of TUNEL positive cells is around renal tubular area due to cisplatin effect, but it is significantly reduced by HP administration. HP alone did not induce any significant tubular cell apoptosis.

Apoptosis can be differentiated from necrosis due to their characteristic nuclear changes. DAPI is a nuclear stain which emits blue fluorescence when excited under fluorescence microscope. DAPI staining was performed for the tissue slices from all groups. Intact cell bodies with clear round nuclei were observed in control group, while weak fluorescence with condensed chromatin and nuclear fragmentation were seen in cisplatin-treated cells compared with control cells (Figure 4). But HP treatment significantly reversed these apoptotic characteristics and recovered the clear nuclear staining at tubular region.

In order to know the molecular mechanism of antiapoptosis of HP, the protein expression of several apoptosis mediators was investigated which includes BCL-2, BCL-xl, Bax, caspase 3, and caspase 7. Treatment of mice with cisplatin did not change the expression of BCL-xL significantly compared to the vehicle-treated normal group (Figures 4(a) and 4(b)), while HP treatment significantly increased the BCL-xL expression, especially at 40 mg/kg dosage, compared to cisplatin model group. Treatment of cisplatin caused higher ratio of Bax/BCL-2 compared to normal group, but HP administration could significantly decrease it in dose-dependent manner compared to cisplatin model group (Figure 4(c)).

In addition, the cleaved caspase 3 and caspase 7 expressions were significantly increased after cisplatin treatment compared to the normal group. HP decreased the cisplatin-induced expression of active caspase 3 and caspase 7 in dose-dependent manner compared to model group (Figure 4(c), *P* < 0.05).

### 3.6. Effect of HP on Cytokine Expression

**Figure 5:**
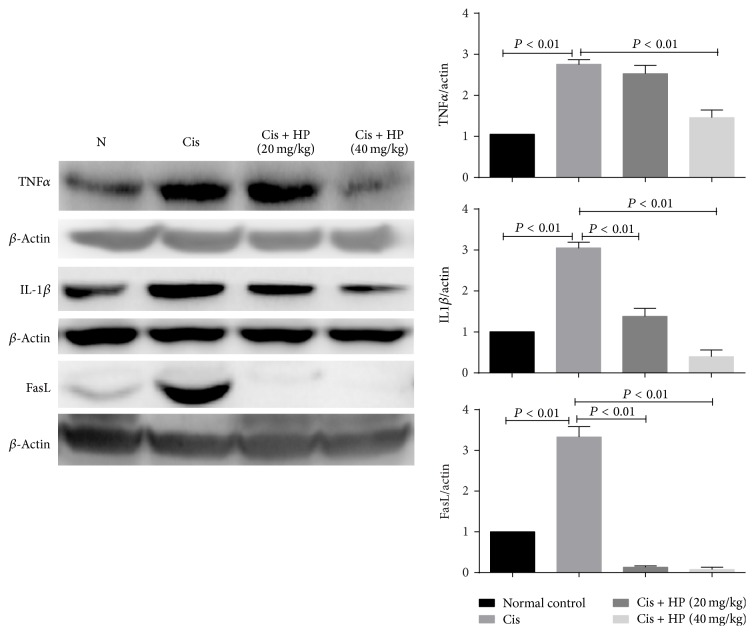
Effect of HP on cytokines expression in cisplatin-induced renal injury in mice by western blot analysis. Data are expressed as mean ± SEM of three independent experiments.

Cisplatin challenge stimulated overexpression of cytokines, and in the current study, we showed a significant increase in TNF*α*, FasL, and IL1*β* in the kidney tissues treated with cisplatin. HP administration markedly suppressed the overexpression of FasL, TNF*α*, and IL1*β* dose-dependently (Figure 5), suggesting that it has potent anti-inflammatory effect.

### 3.7. Effect of HP on Neutrophil and Macrophage Infiltration

**Figure 6:**
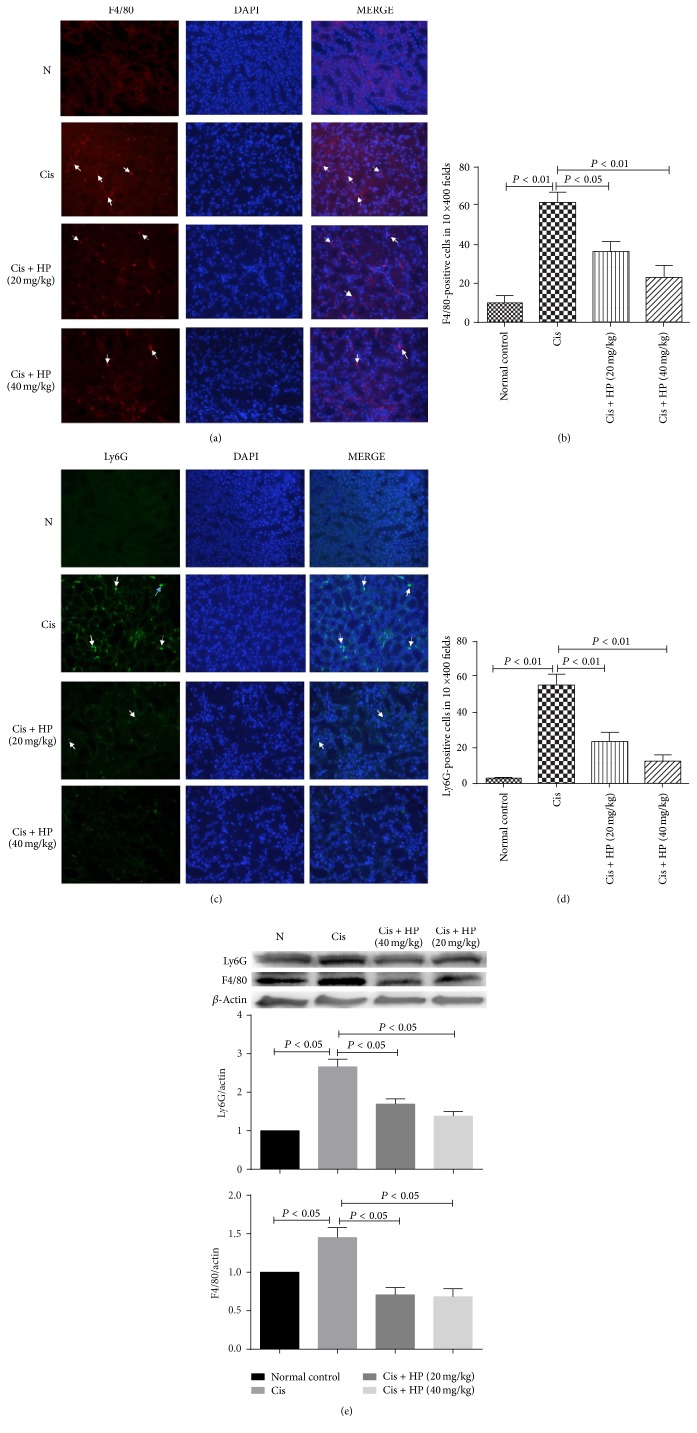
Effect of HP on infiltration of F4/80 positive macrophages and Ly6G-positive neutrophil in cisplatin-induced renal injury in mice. Eight to ten ×400 fields were counted and mean numbers of F4/80 positive and Ly6G positive inflammatory cells were compared at 72 hr after cisplatin administration. (a and b) Representative figures of F4/80 immunofluorescence microscopy and quantitative analysis. (c and d) Representative figures of Ly6G immunofluorescence microscopy and quantitative analysis. (e) Western blot analysis of F4/80 and Ly6G in kidney tissue homogenate. Data are expressed as mean ± SEM of three independent experiments.

Macrophage and neutrophil infiltration is an important pathological characteristic in cisplatin-induced AKI, which was observed by immunofluorescence targeting the biomarkers F4/80 (macrophage) and Ly6G (neutrophil), respectively, in the current study. HP administration could significantly reduce the kidney infiltration of F4/80 positive cells (*P* < 0.01, Figures 6(a) and 6(b)) and Ly6G-positive cells (*P* < 0.05, Figures 6(c) and 6(d)) compared to cisplatin model group. Besides immunofluorescence, western blot analysis using whole kidney tissue lysate also demonstrated that HP could significantly decrease the F4/80 and Ly6G protein level compared to cis model control (Figure 6(e)).

### 3.8. HP Reduced Kidney Inflammation by Downregulating Phosphorylation of p-stat3 and p-Erk1/2

**Figure 7:**
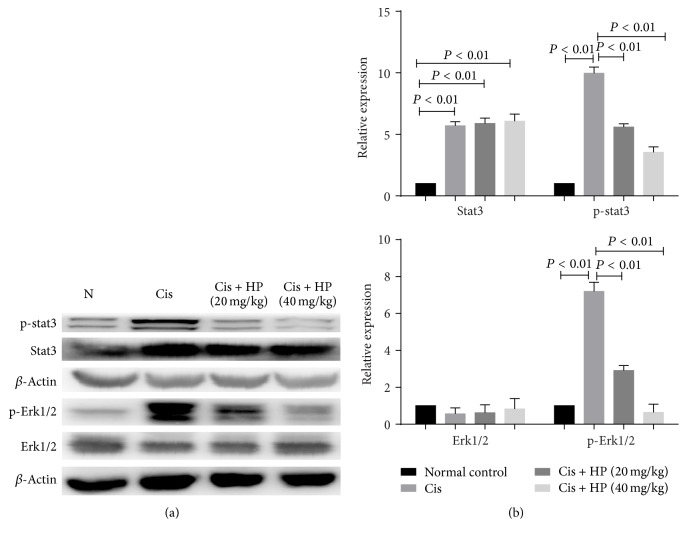
Effect of HP on p-stat3 and p-Erk1/2 in cisplatin-induced renal injury in mice by western blot. Data are expressed as mean ± SEM of three independent experiments.

Overactivation of stat3 and Erk1/2 is two proinflammatory signaling pathways implicated in cisplatin-induced AKI [15, 26]. Stat3 and Erk1/2 phosphorylation plays key role in both signaling pathways. As shown in Figure 7, western blot analysis showed that p-stat3 and p-Erk1/2 were significantly increased at 72 h after injection with cisplatin while administration could significantly downregulate the phosphorylated stat3 and Erk1/2. At the same time, no effective change in the total level of stat3 and Erk1/2 was found.

## 4. Discussion

Cisplatin chemotherapy disadvantage is due to tubulointerstitial inflammation with tubular cell apoptosis, causing acute kidney injury. Therefore, searching for novel therapeutic agent with renal protective effect is highly essential. Traditional Chinese medicine has enormous herbal resources for finding potent medicinal agents.* H. paniculata* is a Chinese herb with anti-inflammation bioactivity, and we have shown in the present study that HP extracts pretreatment reduced the cisplatin-induced AKI by downregulating the renal inflammation and apoptosis.

Cisplatin induces AKI due to its preferential accumulation within the proximal tubular cells in the outer medulla of the kidney [27], thus causing the apoptosis and necrosis in tubular cells. Secondly, increased oxidative stress causes contraction in the mesangial cells, modifications in filtration surface area, and changes in ultrafiltration coefficient factors, thereby leading to decline in the glomerular filtration rate in cisplatin-treated mice [28].

Although several mechanisms contribute to the cisplatin-induced kidney injury, in particular, recent evidence indicates that inflammation plays an important key role in its pathogenesis [29, 30]. Among the abundant cytokines, TNF*α* and IL-1*β* play more important roles in inflammation-mediated renal dysfunctions. Ramesh and Reeves [31] reported that TNF*α* played a central role in the activation of various cytokines, and inhibition of TNF*α* could reduce cisplatin nephrotoxicity effectively [32]. In our present study, we noticed that overexpression of TNF*α* and IL-1*β* induced by cisplatin could be suppressed by HP effectively; thus we think that is one of evidences of HP role in anti-inflammation.

Infiltration of macrophage and neutrophil in the kidney is an important pathogenesis of cisplatin-induced AKI [5]. By immunofluorescence examination, we demonstrated that HP treatment could significantly inhibit such infiltration. A significant increase in the protein levels of macrophage marker F4/80 and neutrophil marker Ly6G at 72 h after cisplatin administration was observed, but HP treatment effectively decreased their levels. Some studies suggest that infiltrating inflammatory cells may act as reservoirs of proinflammatory cytokines and chemokines, which in turn causes these molecules to be released into kidney tissues to cause further injury [12]. Inhibiting the infiltration of inflammatory cells might be one of the other possible mechanisms of HP anti-inflammation.

Apoptosis is now recognized and known as an important mechanism of key player in the cisplatin-induced renal injury. TNF*α* and FasL triggering caspase pathway is one of the major pathways that lead to apoptosis, and subsequent cleaving and activating of caspase 3 and caspase 7 are an another key event for apoptosis occurrence [33, 34]. In the current study, by western blot, we found that HP administration significantly reduced the expression of TNF*α* and FasL, and the cleaved caspases 3 and 7 were significantly reduced. We hypothesized that by reducing the upstream signal that led to direct activation of caspase 3 and caspase 7, HP plays an antiapoptosis effect. On the other hand, HP administration caused higher expression of antiapoptotic BCL-2 BCL-xL and BCL-xL BCL-2 in kidney tissues compared to cisplatin-treated animals, so we consider that this might be another molecular mechanism of HP ameliorating cisplatin-induced tubular apoptosis.

Recent animal studies demonstrated that cisplatin-induced cell death is EGFR-ERK signaling dependent in mouse proximal tubule cells [35, 36], and inhibition of ERK, but not JNK or p38, abolished caspase 3 activation and apoptotic death, suggesting a prodeath role of ERK in cisplatin-induced injury [36]. In the current study, we found HP could significantly inhibit the activation of ERK1/2 by blocking its phosphorylation in a dose-dependent manner. ERK1/2 maybe the upstream signal for TNF*α* production and caspase activation [37]; therefore, we hypothesize that ERK1/2 might be the potential HP target.

Our study clearly indicates the severity of oxidative stress in kidney tissues of cisplatin-treated mice, which was proven by a significant elevation of LPO content and GSH content reduction along with inhibition of SOD and catalase activities in kidney tissues. Coumarin and its derivatives have long been reported to be used as antioxidants [38], and in the current study, we found that HP administration showed an impressive effect on reducing oxidative stress, which demonstrated its antioxidant activity. Meanwhile, elevated cytokine expression and oxidative stress in the kidneys of cisplatin model group were accompanied by increase in p-STAT3 and p-Erk1/2, whereas HP administration suppressed STAT3 and Erk1/2 activation. IL-6/STAT3 pathway has been implicated in renal inflammation [39] and oxidative stress [40, 41]. Therefore, by inhibiting activation of stat3 and Erk1/2, HP might decease the consequent oxidative stress indirectly as well as directly abolish active oxygen ions and superoxide ions.

Skimmin and apiosylskimmin are dominant coumarins from HP, and loganin and sweroside are the main iridoids. Previous study in our laboratory had demonstrated that skimmin has renal protective effect in experimental diabetic nephropathy by inhibiting the activation of TGF*β*1-smad signaling [18] and membranous nephropathy by reducing low-grade inflammation, inhibiting cytokines production and infiltration of lymphocytes [19]. Other researchers also reported that the herb containing skimmin had active anti-inflammation activity by inhibiting the ear swelling caused by xylol of mice [42]. Apiosylskimmin was reported to have antiplasmodial activity [43] and exhibited significant neuroprotective effect against glutamate-induced toxicity [44]. Structure of apiosylskimmin is similar to skimmin which only has one more glucoside, so we guess that apiosylskimmin could be partially metabolized to skimmin in vivo by the liver enzymes to play similar effect as skimmin in vivo. It has been reported that loganin could protect against pancreatitis by inhibiting NF-*κ*B activation [23] and also protect against hydrogen peroxide-induced apoptosis by inhibiting phosphorylation of JNK, p38, and ERK 1/2 in SH-SY5Y cells, which share similar signaling pathway with cisplatin-induced kidney injury [45]. Sweroside can reduce the chlordecone plus CCL4 toxicity to liver and shows significant hepatoprotective effect [46] and sweroside-enriched herbal extract of* Lonicera japonica* could inhibit acetic acid-induced writhing, croton oil and arachidonic acid-induced ear edema, and carrageenan-induced rat hind paw hyperalgesia. Its anti-inflammation mechanisms include inhibition of cyclooxygenase-2 inhibition, inducible nitric oxide synthase, and 5-lipoxygenase activities. These main active ingredients may combine together to form the material basis and may have synergistic effect for anti-inflammation effect.

Previous in vivo studies from our laboratory suggested that administration of extract of* H. paniculata *has better renal protective effect than oral administration of each pure compound from* H. paniculata *at similar dosage, such as skimmin, apiosylskimmin, and loganin (data not shown). Our study indicates that synergistic effect between these molecules may have led to the renal protective effect. So to use a whole natural plant extract is highly beneficial, having different pharmacologically active phytochemicals than an isolated single compound. HP aqueous ethanol extract may exert different pharmacological effects based on one compound and hold different intracellular targets, which function in a synergistic way to enhance the specific activity. Also, the multiple components presence may help in reducing the chance of drug-resistance.

## 5. Conclusions

In conclusion, HP could significantly attenuate cisplatin-induced AKI by improving the renal functions and reducing pathological injuries, and its renal protective effect may derive from its anti-inflammation and antioxidation activities, as well as inhibiting the overactivation of Erk1/2 and stat3.

## Figures and Tables

**Table 1:** Blood urea nitrogen, serum creatinine, and relative kidney weight at the posttreatment with the HP in cisplatin-induced AKI model after five days.

Groups	Normal control	Cisplatin	Cis + HP (20 mg/kg)	Cis + HP (40 mg/kg)	HP (40 mg/kg)
N	8	8	8	8	8
BUN (mg/dl)	29.5 ± 0.72	58.6 ± 6.16^#^	41.56 ± 4.74^*∗*^	32.52 ± 2.71^*∗∗*^	29.88 ± 0.95
Scr (mg/dl)	0.47 ± 0.06	0.73 ± 0.08^#^	0.55 ± 0.07^*∗*^	0.54 ± 0.04^*∗*^	0.46 ± 0.06
Relative kidney weight (%)	0.65 ± 0.029	1.37 ± 0.058	1.18 ± 0.025	1.03 ± 0.053^*∗*^	0.71 ± 0.027

^#^ *P* < 0.05, compared with normal control; ^*∗*^*P* < 0.05 and  ^*∗∗*^*P* < 0.01, compared with cisplatin model group.

**Table 2:** Effect of HP extract on oxidative biomarkers in cisplatin-induced acute kidney injury.

Groups	Normal control	Cisplatin	Cis + HP (20 mg/kg)	Cis + HP (40 mg/kg)	HP (40 mg/kg)
N	8	8	8	8	8
GSH (mmol/g tissue)	8.36 ± 1.04	3.58 ± 0.12^#^	4.73 ± 0.09^*∗*^	5.4 ± 0.36^*∗*^	7.94 ± 0.41
MDA (nmol/g tissue)	44.6 ± 2.42	51.9 ± 2.62	49.6 ± 2.29	47.8 ± 3.52	45.82 ± 4.62
Catalase (U/mg protein)	8.60 ± 0.49	5.62 ± 0.33^#^	6.39 ± 0.44	8.75 ± 0.29^*∗∗*^	8.59 ± 0.58
SOD (U/mg protein)	13.59 ± 0.57	8.66 ± 1.94^#^	11.49 ± 0.94	12.74 ± 0.92^*∗*^	14.53 ± 1.59

^#^ *P* < 0.05, compared with normal control; ^*∗*^*P* < 0.05 and  ^*∗∗*^*P* < 0.01, compared with cisplatin model group.
